# Breast Augmentation: A Cross-Sectional Survey of UK and Irish Aesthetic Surgeons

**DOI:** 10.1093/asjof/ojad070

**Published:** 2023-07-21

**Authors:** Shehab Jabir, Shailesh Vadodaria, Nora Nugent, Thangasamy Kathiresan Sankar

## Abstract

**Background:**

Breast augmentation surgery is the most frequently performed aesthetic surgical procedure within the United Kingdom year on year. However, many variations exist among surgeons regarding various aspects of implant usage and technique.

**Objectives:**

The aim of this study was to evaluate current trends and practices in breast augmentation, within the United Kingdom and correlate them to evidence-based literature.

**Methods:**

An electronic survey of 41 questions was sent to 201 surgeons performing breast augmentation within the United Kingdom and Republic of Ireland. The survey inquired about the surgeons themselves, their practice, implant choice, surgical technique, post-op care, revision surgery, and impact of breast implant–associated anaplastic large cell lymphoma among several other questions.

**Results:**

There were a total of 166 respondents, with 146 completing the survey fully, equaling a response rate of approximately 73%. Overall, there were specific trends in certain aspects such as type of practice, number of augmentations performed per surgeon per year, preferred implant manufacturer, and implant characteristics. That said, there has been a change in other aspects such as implant texture with an increase in the use of smooth implants. The United Kingdom and Ireland concur with certain internationally dominant practice preferences, including the use of inframammary incisions and post-op bra use.

**Conclusions:**

This survey suggests that many aspects of breast augmentation surgery in the United Kingdom are approaching standardization. There are, however, some variations in practice and controversies remaining as expected. It is our belief that further standardizing this very common aesthetic surgical procedure according to evidence-based guidelines will help to improve outcomes for patients.

Breast augmentation is the most frequently performed aesthetic surgical procedure in the United Kingdom, with around 7000 cases reported in 2022.^1^ With the ever-growing repertoire of current breast implants as well as advances in surgical techniques, many variations exist depending on individual surgeon preferences. Some level of consensus does exist both locally within the United Kingdom and internationally with regards to breast augmentation, although the many differences among surgeons outstrip the commonalities. The variety of options with regards to implants includes manufacturer, shape, size/volume, surface texturing, projection, gel cohesivity, etc. In terms of surgical technique, again several choices exist such as pocket plane, incision placement, use of sleeves for implant insertion, intraoperative antimicrobial solutions, use of acellular dermal matrix (ADM) supports, addition of mastopexy, and so on. Apart from implant choice and surgical techniques, other factors not related to the implant and operation itself may impact patient satisfaction and outcomes. These factors include surgeon seniority and type of practice (combination of National Health Service [NHS] and private practice vs full-time private practice), consent process, perioperative and postoperative protocols. The aim of this study was to assess current practices in breast augmentation within the United Kingdom and Ireland and explore them in terms of implant choice, surgical technique, and surgeon demographics, perioperative and postoperative protocols as well as complication rates as reported by individual surgeons. It was hoped that this would uncover areas of consensus while also identifying areas of significant difference in practice where further evidence may help to standardize practice and improve outcomes for patients.

## METHODS

A 41-item, online survey was designed (by the senior author with input from several other senior surgeons), and the questionnaire was sent to surgeons performing breast augmentation within the United Kingdom and Republic of Ireland via a professional survey instrument (SmartSurvey, Gloucestershire, UK). Surgeons surveyed were members of the British Association of Plastic, Reconstructive and Aesthetic Surgeons (BAPRAS), and British Association of Aesthetic Plastic Surgeons (BAAPS) and contacted via e-mail correspondence or WhatsApp messaging (Menlo Park, CA). The survey addressed 7 areas of interest: surgeon/member demographics and practice patterns, implant factors, surgical techniques, perioperative and postoperative care, technical considerations in revision procedures, personal perception of complications, and impact of breast implant–associated anaplastic large cell lymphoma (BIA-ALCL) on current practice. It was launched on March 23, 2021 and closed on July 25, 2021 with 2 reminders sent to surgeons to complete the survey at approximately 6 week intervals. Responses were anonymous and the results were tabulated using Microsoft Excel (Microsoft Corp., Redmond, WA). Questions that included a commentary option were studied individually to uncover relevant issues potentially overlooked by question design.

## RESULTS

Out of a total of 201 surgeons to whom the survey was sent, 166 responded. Out of the 166 respondents, 146 completed all questions on the survey resulting in a response rate of 72.6%. Tables 1–7 show respondents’ answers to questions posed in the survey.

**Table 1.** Member Demographics and Practice Patterns

Characteristic	% of respondents
Area of surgical practice	
Plastic and reconstructive surgeon	95.21
Breast surgeon	3.42
Unknown	1.37
Type of practice	
NHS and private sector	65.07
Private sector only	32.88
NHS only	2.05
Years in practice	
0-5	18.49
5-10	19.18
>10	62.33
Annual number of primary augmentations	
0-25	39.73
26-50	31.51
51-100	18.49
>100	10.27

NHS, National Health Service.

### Member Demographics and Practice Patterns

Most practitioners performing breast augmentations were plastic surgeons (95.21%), and a much small number of implants were inserted by general surgery trained breast surgeons (Table 1). Most surgeons had a mixed practice which combined between the NHS and private practice (65.07%), although a third of surgeons were in full-time private practice (32.88%). A very small number practiced only within the NHS. In terms of years in practice, there were 3 time points in the survey with almost two-thirds being in practice for over a decade (62.33%).

### Implant Factors

Despite the availability of a large range of implant manufacturers within the UK market, UK practice is clustered around 3 main manufacturers, namely Mentor (Irvine, CA), Nagor (GC Aesthetics; Glasgow, UK), and Motiva (Establishment Labs Holdings Inc.; Alajuela, Costa Rica; Table 2). Most patients and surgeons chose round implants over anatomically shaped implants for primary breast augmentation. In terms of texturing, textured implants are still the preferred implant by most surgeons with over 80% choosing some types of textured implant over a smooth implant. The type of texturing, however, was almost equally distributed between macro, micro, and nano texturing. The most commonly utilized size range was 250 to 350 cc implants with much smaller numbers being utilized at either end of the size spectrum, that is <250 cc and above 450 cc. Finally, ADMs were not utilized by most surgeons, although some would consider using them in selected cases of breast augmentation mostly related to congenital or acquired deformities.

**Table 2.** Implant Factors

Factor	% of total respondents
Currently I use the following implant manufacturers (multiple responses allowed)	
B Lite (Polytech Health & Aesthetics GmbH, Dieburg, Germany)	6.85
Eurosilicone (GC Aesthetics; Apt, France)	4.79
Ideal (saline) (Ideal Implant Incorporated, Addison, TX)	0.00
Mentor (Johnson & Johnson, Irvine, CA)	71.23
Motiva (Establishment Labs Holdings Inc., Alajuela, Costa Rica)	27.40
Nagor (GC Aesthetics, Glasgow)	27.40
Polytech (Polytech Heath & Aesthetics GmbH, Dieburg, Germany)	16.44
Sebbin (Groupe Sebbin, Paris, France)	12.33
Sientra (Sientra Inc., Santa Barbara, CA)	0.00
Silmed (polyurethane) (Silimed Industria de Implantes Ltda; Rio de Janeiro, Brazil)	0.68
Others	0.68
The most common implant that I use in my practice	
B Lite	0.00
Eurosilicone	0.00
Ideal (saline)	0.00
Mentor	56.16
Motiva	19.86
Nagor	11.64
Polytech	3.42
Sebbin	5.48
Sientra	0.00
Silmed (polyurethane)	0.00
Others	3.42
Patient's choice of implant for primary augmentation	
Anatomical	13.01
Round	78.08
Other (please specify)	8.90
Surgeons’ choice of implant for primary augmentation	
Anatomical	14.38
Round	75.34
Other (please specify)	10.27
Textured vs smooth implants; I use the following implant	
Smooth	16.44
Macro textured	28.08
Micro textured	28.77
Nano textured	23.29
Other (please specify)	3.42
Size of implant, cc	
150-250	2.74
250-350	80.82
350-450	15.75
>450	0.68
Acellular dermal matrix as an implant stabilizer	
Do not use it at all	83.33
Use it in selected cases	14.58
Use it in all cases	0
Other (please specify)	3.47

### Surgical Techniques in Primary Augmentation

The inframammary fold (IMF) incision for implant placement was by far the commonest incision employed (Table 3). In terms of pocket plane, the subglandular pocket was used slightly more preferentially compared to submuscular pocket location. Within the submuscular pocket location, the dual plane technique as described by Tebbett was the most commonly utilized technique. Most surgeons performed pocket dissection with electrocautery, performed a rinse of the implant and/or pocket with iodine (betadine), and used a sleeve for implant insertion.

**Table 3.** Surgical Techniques in Primary Augmentation

Surgical technique	% of total respondents
Incision	
Infra mammary incision	99.32
Trans-axillary incision	0.68
Periareolar incision	1.37
Other (please specify)	0.68
Pocket	
Submuscular	42.47
Subglandular	57.53
If submuscular, I prefer	
Traditional subpectoral dissection	6.85
Dual plane (Tebbetts)	80.14
Biplane	6.16
NA	3.42
Other (please specify)	3.42
My surgical dissection technique includes	
Blunt finger dissection	2.74
Total electro dissection	69.86
Sharp dissection with scissors or knife	1.37
Combination method of blunt and electro dissection	33.56
I use the following to rinse the implant and/or pocket	
Iodine (betadine)	50.68
Triple antibiotic solutions	19.18
Saline	7.53
None	2.05
Other	20.55
Use of sleeve for implant insertion	
Yes	62.33
No	37.67

NA, not applicable.

### Perioperative and Postoperative Care

In terms of postoperative dressings, most surgeons used steristrips only on the suture line postoperatively (Table 4). However, there are a wide variety of preferences in this regard with several different options. Most patients had a single intraoperative intravenous (IV) antibiotic dose, although a significant number of surgeons provided postoperative oral antibiotics for a period of 5 to 7 days following primary breast augmentation. Most surgeons also gave patients an intraoperative dose of IV tranexamic acid to stem any bleeding. With regards to deep venous thrombosis (DVT) prophylaxis, most surgeons relied on thromboembolic deterrent (TED) stockings, intraoperative intermittent pneumatic compression calf pumps together with early mobilization, and adequate hydration to reduce the likelihood of a DVT. Drains were only used by a minority (10%) with most practitioners preferring not to use surgical drains. A support bra in the immediate postoperative period was utilized by over 80% of surgeons. When it came to preoperative mammography for patients over 40 years of age irrespective of family history, there was an almost 50-50 divide, with half requesting it and the other half proceeding to surgery without a mammogram.

**Table 4.** Perioperative and Postoperative Care

Characteristic	% of total respondents
Dressing of choice on suture line	
Prineo tape and glue	3.42
Only glue	16.44
Steristrips	58.22
Mepore	23.29
Opsite	19.18
Other (please specify)	21.92
Prophylactic systemic antibiotics	
I do not use antibiotics	2.05
One shot IV intraoperatively only	44.52
One shot IV intraoperatively followed by two IV postoperative doses	15.07
One shot IV intraoperatively and a further 5 to 7 days of oral antibiotics	38.36
Use of tranexamic acid	
IV dose intraoperatively	55.48
Into the pocket	6.85
Only if there is significant bleeding	21.23
No	22.60
My preferred choice of DVT prophylaxsis	
TED stockings	89.04
Flowtron boots (Arjo Inc., Addison, IL) (intraoperative only)	57.53
Flowtron boots (intraoperative and postoperative period)	29.45
Early mobilization	84.93
Adequate hydration	69.18
Chemo prophylaxsis	15.75
Other (please specify)	5.48
Postoperative surgical bra	
Routinely use it during immediate post-op period	83.56
Only after all dressings are off	8.22
Patient's choice	4.11
Other (please specify)	4.11
Drains	
Yes	10.96
No	89.04
Routine breast screening for those over 40 irrespective of family history	
Yes	50.68
No	49.32

DVT, deep vein thrombosis; IV, intravenous; TED, thromboembolic deterrent.

### Technical Considerations in Revision Procedures

Most surgeons very rarely or rarely (92.36%) performed revision augmentation for capsular contracture within 5 years of a primary augmentation (Table 5). Implant choice for revision procedures was almost overwhelmingly round (92%) with 8% choosing an anatomical implant for a revision procedure. In most cases, the implant was removed followed by a total capsulectomy (46.4%), while an en-bloc technique was only employed 8.5% of the time. In cases of significant capsular contracture, most surgeons (50.7%) preferred to change the pocket plane, while a reasonable number still placed the implant within the same pocket (37.9%). Surgeons were also surveyed about a concomitant mastopexy at the time of revision augmentation and whether this impacted pocket location. In cases where revision augmentation was accompanied by a mastopexy, 45% used a submuscular pocket, 39% used a subglandular pocket, and 11.4% of surgeons changed the plane/location of pocket altogether (Table 5). In terms of implant texturing for revision cases, again a wide variety of textured implants were utilized with smooth implants being used less commonly. The most common complications encountered in revision surgery were hematoma and loss of nipple sensation.

**Table 5.** Technical Considerations in Revision Procedures

Technical considerations	% of total respondents
Revision for capsular contracture within 5 years of primary breast augmentation	
Very rarely	72.92
Rarely	19.44
Neither frequent nor rarely	5.56
Frequently	1.39
Very frequently	0.69
Implant choice for revision surgery	
Anatomical	7.86
Round	92.14
In revision surgery for capsular contracture or rupture:	
Always do a total intact capsulectomy (enbloc removal; implant in situ with entire capsule)	8.57
Always do a total capsulectomy but not always an en-bloc removal	46.43
Partial capsulectomy	22.86
Only capsulotomy	1.43
Other (please specify)	20.71
In significant capsular contracture revision surgery	
Only change the plane if mastopexy is needed	11.43
Change the pocket plane	50.71
Replace into the same pocket	37.86
If revision surgery is accompanied by an uplift, I most commonly use	
Submuscular pocket	45.00
Subglandular pocket	39.29
Other	15.71
Most common implant texture for revision surgery	
Smooth	11.43
Textured	27.14
Nano textured	20.71
Micro textured	33.57
Polyurethane coated	3.57
Fat graft instead	0
Other (please specify)	3.57

Most common complication I have encountered in revision surgery	Most commonly (%)	Frequently (%)	Rarely (%)	Very rarely (%)	Not seen (%)
Hematoma	7.1	2.9	20.7	45.7	23.6
Seroma	0	0.7	17.1	40.7	41.4
Infection	0.7	0.7	17.1	43.6	37.9
Re-encapsulation	0.7	2.1	23.6	29.3	44.3
Rippling	0.7	10.7	30.0	35.7	22.9
Implant too big or too small	0.7	5	34.3	40.0	20.0
Nipple malposition	1.4	7.9	38.6	30.7	21.4
Loss of nipple sensation	2.9	17.1	41.4	24.3	14.3

### Personal Perception of Complications

Surgeons were also surveyed about their complications following primary breast augmentation. The vast majority of surgeons reported that they had a post-op hematoma rate of <1%. DVTs were almost never seen by this cohort of surgeons (Table 6). Fifty-nine percent never had a postoperative infection and 31% had a <1% postoperative infection rate.

**Table 6.** Personal Perception of Complications

Complication	% of total respondents
Within the last 5 years of practice my rate of immediate post-op hematoma was	
<1%	80.56
1%-5%	13.89
5%-10%	0
>10%	0.69
Other (please specify)	4.86
In the last 5 years I have encountered a post-op DVT	
Yes	0.69
No	99.31
In the last 5 years, I have had post-op infections (requiring extended antibiotics or readmission)	
None	59.03
<1%	31.94
Between 1% and 5%	8.33
>5%	0
Other	0.69

DVT, deep vein thrombosis.

### Impact of BIA-ALCL on Current Practice

Some aspects of the impact of BIA-ALCL on breast augmentation practice were also surveyed (Table 7). Most surgeons altered their practice by changing to a different implant, either a different type of textured implant or a smooth implant. Intriguingly, one-third felt that the recent events related to breast implants and BIA-ALCL did not alter their practice. Even more interesting was that some practitioners were considering stopping breast augmentation altogether, while a few had already done so following the recent scientific developments and media attention related to BIA-ALCL. That said, most surgeons had not yet come across a case of BIA-ALCL in their practice (83.33%). Despite the small risk of BIA-ALCL, most surgeons reported that most patients were still willing to proceed with breast augmentation surgery.

**Table 7.** Impact of ALCL on Current Practice

Impact	% of total respondents
In the last 5 years, the increased media and scientific research on ALCL has	
Not changed my practice	33.33
Changed to using more fat grafting	2.08
Changed to a different implant maker	38.19
Changed to a different texture or smooth implant	42.36
Considering stopping breast augmentation surgery	6.94
Stopped doing breast augmentations	1.39
In the last 5 years I have seen and/or treated	
1-5 patients	15.97
5-10 patients	0.69
Over 10 patients	0
None	83.33
Other (please specify)	0
Following information given regarding ALCL	
Most patients are not keen to proceed with surgery	0.69
Only a small number are not keen to proceed to surgery	6.94
Most of them are keen to proceed with surgery	92.36

ALCL, anaplastic large cell lymphoma.

## DISCUSSION

As the most performed cosmetic surgery intervention within the United Kingdom and internationally, breast augmentation and its technical refinements are undergoing constant evolution. Several considerations exist and techniques are in a regular state of evolution. This study aimed to capture a snapshot of current practice among United Kingdom and Republic of Ireland surgeons performing breast augmentation.

Breast augmentation is considered a predominantly cosmetic intervention within the United Kingdom and as such is not performed within the NHS nor is it funded within the private sector by health insurance. Thus, most breast augmentations are undertaken by self-paying patients in the private sector. That said, it is possible that patients with significant breast asymmetry or congenital breast deformity may obtain individual funding approval from clinical commissioning groups to have implant-based corrective surgery to reconstruct their breasts. This is a very limited group due to funding and approval constraints within the public sector. Postbreast cancer symmetrizing breast augmentation also takes place within the public system. This explains the small number of implant augmentations (2.05%), occurring among surgeons practicing purely within the NHS. In the United Kingdom and Ireland, breast surgery is performed primarily by 2 groups, breast surgeons (general surgery trained with a specialist interest in breast surgery including breast oncological surgery) and plastic surgeons. Plastic surgeons continue to dominate the field of aesthetic breast surgery with over 95% of breast augmentation surgery being performed by them. There is a current initiative in the United Kingdom to certify cosmetic surgical practice via index numbers of procedures undertaken in various fields such as cosmetic breast surgery, cosmetic facial surgery, and so on along with other evidence of ethical and knowledgeable practice in aesthetic surgery.^2^ This may impact who carries out aesthetic breast surgery in the future. In this study, most practitioners of breast augmentation surgery performing <25 such cases per annum still appeared to maintain high standards with minimal postoperative complications.

Although several aspects of implant choice could have been examined in the study, we restricted questions related to implants to what we felt were the most important ones for surgeons. It was clear that the market leader in terms of manufacturers of implants within the surgeon group surveyed was Mentor implants. Round implants continue to dominate over anatomically shaped implants both in terms of patients’ and surgeons’ choice. Although the exact reasons for this were not explored further in the study, we feel that concerns around potential implant malposition or rotation, the lack of studies proving aesthetic superiority of anatomical implants over round implants, their higher cost and patients’ requests for round implants may be some of the factors that result in surgeons choosing round implants over anatomically shaped implants. There are also some differences in surgical placement between the two. Textured implants continue to dominate usage in the United Kingdom despite concerns regarding BIA-ALCL, although the type of textured implant has evolved with more micro- and nano-textured implants being increasingly utilized over standard texturing. That said, smooth implant usage has continued to rise, and it may be that as further evidence of BIA-ALCL and its prevalence in textured implants continues to be elucidated, smooth implant usage within the United Kingdom will continue to grow. The most utilized implant size range as expected within the surveyed surgeons is between 250 and 350 cc with implant sizes that are smaller and larger than this range being utilized less frequently. Most UK patients want a “natural” looking increase in their breast volume. This, together with UK practitioner's prioritization (in most cases) of what a patient's tissue can “take” (tissue-based planning) over “what they want” has led to a decreasing utilization of larger implant volumes.

The commonest incision utilized in the survey for implant placement was within the IMF crease, which concurs with previous national and international surveys.^3,4^ This incision provides good access to the breast for implant insertion, is hidden inconspicuously within the breast crease, and is easy to master. In terms of pocket location, there was an almost 50-50 split among surgeons performing subglandular vs submuscular pockets. The subglandular approach has a number of advantages including an easier dissection plane with less bleeding, less postoperative pain and avoids implant deformation that may occur with animation of muscle in the submuscular plane. That said, it is also associated with higher capsular contracture rates, is not recommended in patients with inadequate soft tissue cover as it increases the chances of visible wrinkling and rippling of the implant, and provides less support and stabilization of the implant compared to the submuscular location. The submuscular location on the other hand has lower capsular contracture rates, provides good coverage of the implant which in turn reduces the possibility of visible wrinkling and rippling of the implant, provides enhanced support of the implant, and enables enhanced imaging of the breast on mammograms.^5,6^ It, however, also has disadvantages which include animation deformity, increased postoperative pain, and increased risk of overlying breast ptosis over time with the “waterfall deformity” occurring. In terms of the various submuscular techniques available, the dual plane technique as described by Tebbetts was by far the most commonly utilized.^6^ However, the exact type of dual plane technique (Type 1, 2, or 3) employed was not queried within the survey. Pocket dissection was performed exclusively via electrocautery by most. This enables easy dissection of the plane due to clear visualization of the pocket plane and the ability to cauterize bleeding vessels concurrently and prospectively. Some utilize a combination of blunt dissection together with electrocautery while a small number used finger dissection. Finger dissection is a more blind technique and liable to create unnecessary bleeding with poor localization of the pocket plane. Despite the lack of clear evidence to support the utility of implant and pocket rinsing with a variety of antimicrobial agents from iodine to antibiotic solution and saline, surgeons continue to do this in the belief that it may reduce the risk of infection.^7‐10^ Sleeves for implant insertion are also being increasingly utilized. This may be partly due to the wider availability of sleeves but also because they have other advantages such as easier insertion of larger implants, reducing implant and tissue trauma while inserting manually with digits and reducing skin contact and exposure time of the implant. This again, however, does not have clear scientific evidence of reducing infection risk which could be classified as another proposed benefit.^11‐13^

A wide variety of preoperative, perioperative, and postoperative practices for patients with breast augmentations were also surveyed. In the United Kingdom, breast cancer screening for females without any family history of breast cancer starts at age 50. Typically, an invite from the NHS breast screening service is sent for breast screening between the ages of 50 and 53, and further breast screening is performed every 3 years until the age of 70.^14^ Females who have a moderate or high risk of breast cancer because of their family history will have screening mammograms every year from the age of 40 onwards, while those younger than 40 with a high risk of developing breast cancer are offered MRI scans from the age of 30 onwards. Our survey noted that over half of the surgeons surveyed (50.7%) requested screening mammograms for patients 40 years or older irrespective of family history of breast cancer in line with joint guidance issued by the British Society of Breast Radiology, Association of Breast Surgeons, and the British Association of Plastic Reconstructive and Aesthetic Surgeons.^15^ The remainder (49.3%) did not request screening mammograms.

In terms of antibiotic usage, there is evidence that suggests that a single dose of IV antibiotics is sufficient as prophylaxis in primary breast augmentation surgery, while the extra duration of antibiotics post-op does not result in reduced superficial or periprosthetic infections.^16^ Although infection in primary breast augmentation surgery is a rare occurrence, where an infection does develop, it is usually a challenging problem to manage. Tranexamic acid has been utilized in a number of surgical domains, especially within the realm of trauma surgery, to reduce blood loss. Following its successful utilization in this domain, almost all other surgical specialties with the risk of a reasonable degree of blood loss (and in some cases areas where there might in fact be very little blood loss!) have started incorporating a dose of IV tranexamic acid either IV or within saline solution as a topical agent or both. Although there is a lack of evidence of its benefit specifically within breast augmentation surgery, there is evidence from implant-based breast reconstruction as well as breast surgery and within the wider plastic surgery literature that shows a clear benefit of using tranexamic acid to decrease blood loss regardless of the administration route, with no increased risk of thrombosis events.^17‐19^ Tranexamic acid also elicits a potent anti-inflammatory response with a decrease in postoperative edema and ecchymosis, which improves recovery time.^20,21^ Preferences with regards to DVT prophylaxis were also examined. Most surgeons relied primarily on TED stockings, early mobilization, use of intermittent pneumatic compression boots intraoperatively and adequate hydration to reduce the risk of DVT. Chemoprophylaxis was much less commonly utilized and probably reflects the fact that most patients undergoing breast augmentation are younger, fit, and active females who can start mobilizing almost immediately after surgery because it is usually carried out as day-case surgery.

In terms of postoperative dressings, there was a wide variety of dressings used, although steristrips appear to be the most commonly utilized. Most surgeons utilize a postoperative support bra immediately following surgery and do not use drains. Although there is no clear evidence in the literature to support either of them in primary breast augmentation surgery, there is evidence from aesthetic secondary implant exchange surgery that suction drain use in these cases is associated with an increased risk of surgical-site infection. Surgeons should therefore carefully consider using suction drains in selected cases only.^22^

Secondary revision procedures are an inevitable consequence of breast implant augmentation in most patients. Capsular contracture is the commonest reason for revision surgery with other reasons such as implant malposition and size change reported as being less common reasons for secondary surgery. A plethora of factors influence the development of capsular contracture, the most important of which have been shown to include surface texturing (textured implants have less capsular contracture vs smooth implant), pocket location (submuscular pockets tend to produce less capsular contracture vs subglandular), and implant fill (saline-filled implants produce less capsular contracture than silicon filled implants).^23^ The time point at which capsular contracture develops has been variously mentioned within the evidence as occurring at weeks to months following implant placement and becoming well established at approximately 2 years following insertion.^24‐26^ That said, clinically significant capsular contracture requiring revision surgery may occur much later in the vast majority of patients.^24‐26^ This is evidenced by over 90% of the surgeons surveyed having either very rarely or rarely dealt with a capsular contracture up to 5 years postsurgery. For revision surgery, most surgeons prefer to use round, textured implants. The capsule is most commonly dealt with via a total capsulectomy, and half of surgeons surveyed changed the pocket location in revision surgery. These steps all appear to concur with evidence that suggests capsular contracture may be reduced via these steps in revision cases.^27,28^

Hematoma is probably the most common immediate complication following breast augmentation surgery. Although the surgeon-reported hematoma rate is a much less accurate figure than that may be obtained, for example via an audit of one's own practice or operating room records, it does provide some idea of the rate at which this complication occurs among individual surgeons. The reported hematoma rate for most surgeons is in line with hematoma rates following breast augmentation provided in the literature. Infection which again may occur in the early postsurgery phase, although uncommon, can be among the most challenging complications to deal with. Over 90% of surgeons had no infections or felt their infection rates were <1% over a period of 5 years which again is within the accepted limits. Finally, as the duration of breast augmentation surgery is short and the majority of patients belong to ASA Grade 1, mechanical prophylaxis seems to be more than adequate to prevent thromboembolic incidents.

Since the discovery of BIA-ALCL as a clinical entity following breast augmentation (particularly with textured implants), there has been an enormous impact on the practice of surgeons performing breast augmentation both nationally and internationally.^29‐31^ That said, within the surveyed surgeons, it appears BIA-ALCL has not had a significant impact on practice of approximately one-third of surgeons, while slightly higher numbers have either changed the implant manufacturer they use (38%) or switched to a different texture or smooth implant altogether (42.4%; Table 7). A smaller number have considered stopping performing breast augmentation with implants altogether and a very small number have in fact stopped. There was no comparable data found in the published literature on the impact of BIA-ALCL on individual surgeon's practice. Reassuringly, most surgeons have not come across a case of BIA-ALCL over 5 years of practice and only a small number of patients appear to be put off from performing primary aesthetic implant-based breast augmentation following a discussion of the risks of BIA-ALCL.

While this survey provided several key findings with regards to breast implant usage and surgeon practice within the United Kingdom, it also has several limitations. One of the primary limitations lies in the design of the study as an electronically disseminated survey/questionnaire. Despite having a high response rate of 72.6% which is considered an important statistic for judging the quality of a survey, several authors have questioned its validity as a research method in and of itself.^32^ Still, surveys enable one to obtain a quick and useful snapshot of current trends prevailing within the area of study. In addition, another limitation is the number of questions incorporated into the survey. A total of 41 questions could mean that only surgeons with an interest in this domain persisted with completing the questionnaire and therefore self-selected themselves to respond to the survey. A third limitation is that several of the questions, especially ones regarding complication rates are subject to recall bias. It is very likely that most surgeons could not remember all their complications and even if they could, it would be difficult for them to arrive at a personal complication rate over a period of 5 years unless the data were collected and specifically audited by them. In addition, despite the survey enabling surgeons to record practices that deviated from the options provided within the survey by giving them an “other” free text option, most surgeons may have picked an option from the survey for the sake of convenience and saving time. Finally, no outcome measures were analyzed. It was assumed that the surgeon's choice of a particular technique yielded the best result in their hands which, could very well not be the case.

## CONCLUSIONS

There appears to be concordance among many aspects of breast augmentation between surgeons in the United Kingdom and Republic of Ireland as may be expected from a study confined to one geographical location. That said, there are still a handful of factors that the surveyed surgeons are divided upon with equal numbers on either side of the issue. As new evidence evolves and is published and nuances to the established techniques are introduced to what is essentially the most common cosmetic procedure in the United Kingdom and internationally, plastic surgeons involved in breast augmentation may still benefit from continued research within this domain in order to create standardized evidence-based practice guidelines and further improve outcomes for patients.
